# Interactions of Non-Nutritive Artificial Sweeteners with the Microbiome in Metabolic Syndrome

**DOI:** 10.20900/immunometab20220012

**Published:** 2022-04-18

**Authors:** Valerie Harrington, Lilian Lau, Alexander Crits-Christoph, Jotham Suez

**Affiliations:** W. Harry Feinstone Department of Molecular Microbiology and Immunology, Johns Hopkins Bloomberg School of Public Health, Baltimore, Maryland 21205, USA

**Keywords:** microbiome, non-nutritive artificial sweeteners (NAS), metabolic syndrome, obesity, diabetes, metagenomics, metabolomics

## Abstract

Replacing sugar with non-nutritive artificial sweeteners (NAS) is a popular dietary choice for the prevention and management of metabolic syndrome and its comorbidities. However, evidence in human trials is conflicted regarding the efficacy of this strategy and whether NAS may counterintuitively promote, rather than prevent, metabolic derangements. The heterogeneity in outcomes may stem in part from microbiome variation between human participants and across research animal vivaria, leading to differential interactions of NAS with gut bacteria. An increasing body of evidence indicates that NAS can alter the mammalian gut microbiome composition, function, and metabolome, which can, in turn, influence host metabolic health. While there is evidence for microbiome-mediated metabolic shifts in response to NAS, the mechanisms by which NAS affect the gut microbiome, and how the microbiome subsequently affects host metabolic processes, remain unclear. In this viewpoint, we discuss data from human and animal trials and provide an overview of the current evidence for NAS-mediated microbial and metabolomic changes. We also review potential mechanisms through which NAS may influence the microbiome and delineate the next steps required to inform public health policies.

## NON-NUTRITIVE ARTIFICIAL SWEETENERS AND METABOLIC SYNDROME RISK

Non-nutritive artificial sweeteners (NAS), including saccharin, sucralose, aspartame, neotame, cyclamate, and acesulfame potassium (AceK), are an increasingly popular dietary choice among children [1,2] and adults [1,3], as they maintain the sweet taste of foods and beverages but, unlike sugar, they do not contain calories nor do they elicit a post-prandial increase in blood glucose levels. For this reason, health authorities often recommend substituting caloric sweeteners with NAS for the management and prevent metabolic syndrome and associated morbidities, including diabetes, stroke, and cardiovascular disease [4]. Nonetheless, the prevalence of obesity and diabetes continues to increase globally [5], fueling concerns that NAS may, in fact, contribute to the metabolic syndrome pandemic [6]. Both retrospective cross-sectional studies and prospective cohort trials indicate an association between NAS intake and elevated risk of metabolic syndrome [6,7] and its associated morbidity [6,8–10]; however, these studies do not provide causal evidence and may be misinterpreted due to reverse causality. Interestingly, some NAS (saccharin, sucralose, and AceK) can be detected in amniotic fluid, cord blood, and breast milk [11,12], and several [13–16], but not all [17] studies associate maternal NAS intake during pregnancy or breastfeeding and elevated BMI and adiposity of the offspring, even when controlling for maternal BMI or diet quality. Evidence from this type of exposure may be more compelling as it is less prone to reverse causality [18], although additional factors, such as maternal genetic predisposition to obesity, may underlie these associations.

While intervention and specifically randomized-controlled trials (RCTs) could provide more rigorous evidence for either a protective or detrimental effect of NAS on metabolic health, their results thus far have been inconclusive. Several intervention trials suggest a causal link between NAS consumption (predominantly sucralose and saccharin) and worsened glucose tolerance [19–23], whereas others report neither a detrimental nor beneficial effect [24,25]. The effect of NAS on body weight is also conflicted, with some reporting facilitated weight loss [26–30] and others sweetener-dependent (saccharin) weight gain [31]. Consequently, meta-analyses of RCTs are inconclusive and may not support the desired beneficial effects of NAS on metabolic health [6,32–34].

Notably, there is considerable heterogeneity between trials in the studied cohorts (adults vs children and adolescents, individuals with or without metabolic syndrome, obesity, or diabetes) and methodology (type and dose of NAS, duration of exposure, NAS administered with carbohydrates or in pure form, comparison to consumption of caloric sweetener or no supplement). Coupling the supplemented NAS with carbohydrates such as glucose or maltodextrin, which are present in commercial NAS sachets as fillers, may contribute to a negative effect associated with NAS in some studies [20–22] but not others, which used purified forms of NAS [24,25,35,36]. Nonetheless, longer exposure to NAS is associated with a negative effect on metabolic health even in the absence of carbohydrates [19,23,31].

Some of the factors that contribute to heterogeneity in human nutrition trials [37], including habitual diet (and whether it already contains NAS, sometimes unknowingly [1]), effective blinding, compliance, and defining appropriate control groups, may be circumvented by NAS feeding trials in animal models. Multiple such studies, mostly in rodents, causally link between NAS supplementation and impaired metabolic health [20,38–53], although some variation still exists, even in model systems [35,54–57].

## INTERACTIONS BETWEEN NAS AND THE INTESTINAL MICROBIOME IN METABOLIC SYNDROME

An emerging factor that likely underlies some of the heterogeneity between trials and could potentially be used to resolve conflicting reports is the intestinal microbiome. This dense community of microorganisms that naturally reside in the gastrointestinal tract plays key roles in mammalian metabolic health and disease [58] and in mediating the effects of nutrients [59] and dietary supplements on metabolic health [60]. Notably, the microbiome configuration displays considerable person-to-person heterogeneity and differs between mice obtained from different suppliers or housed in different research vivaria. This variability is associated with personalized responses to diets [61–65] and therapeutics [66–70], as well as opposing phenotypes in animal studies [71–73]. Furthermore, presence of sucralose [46,52,74], saccharin [35,75], and AceK [46,52] has been demonstrated in stool samples from NAS-supplemented animals and human subjects. Thus, it is plausible that NAS interact with the intestinal microbiome, which can translate to an effect on the mammalian host. Consequently, variation in microbiome configurations between human cohorts and animal vivaria may result in differential NAS-microbiome interactions and downstream health outcomes.

The majority of evidence for NAS effects on the intestinal microbiome stems from feeding trials in animal models. To provide an overview, we searched PubMed for original research (excluding reviews and meta-analyses) in mammals (excluding humans discussed separately), focusing exclusively on NAS (saccharin, sucralose, aspartame, AceK, neotame, cyclamate) in combination with the keyword “microbiome” or “microbiota”. This resulted in **28 trials** showing an effect of NAS on the mammalian microbiome across 49 different experimental conditions (sex, dose, NAS formulation, diet, age) [20,35,38–40,46,48,52,76–96], and **only four trials** (six arms) that report no such effect [35,52,82,96] (Table 1). Even when considering the difficulty in reliably validating null results (and publishing them), these studies provide strong evidence that different types of NAS can alter the mammalian microbiome in a range of doses, background diets, administration modes, and duration of exposure (Table 1). Notably, modulation of the microbiome does not necessarily indicate an effect on host health; however, many of these studies associate an effect of NAS on the microbiome with a negative impact on the host’s metabolic health (Table 1). Two of these studies further provide a causal link between NAS-induced microbiome alterations and worsened metabolic health by demonstrating glucose intolerance manifesting in germ-free (GF) mice receiving microbiome from saccharin-exposed mice [20] or glucose intolerance coupled with weight gain and adiposity in GF mice receiving microbiome from rat offspring of aspartame-drinking dams [78].

The aforementioned works demonstrate that NAS can profoundly impact the mammalian microbiome, with numerous bacterial taxa reportedly increasing while others decrease following exposure to NAS. This observation refutes the notion that NAS are inert and raises several important questions: first, what are the mechanisms through which NAS reshape the microbiome? How does the interaction between NAS and the microbiome disrupt the host’s metabolic health? And are similar effects observed in humans?

**Figure 1.**
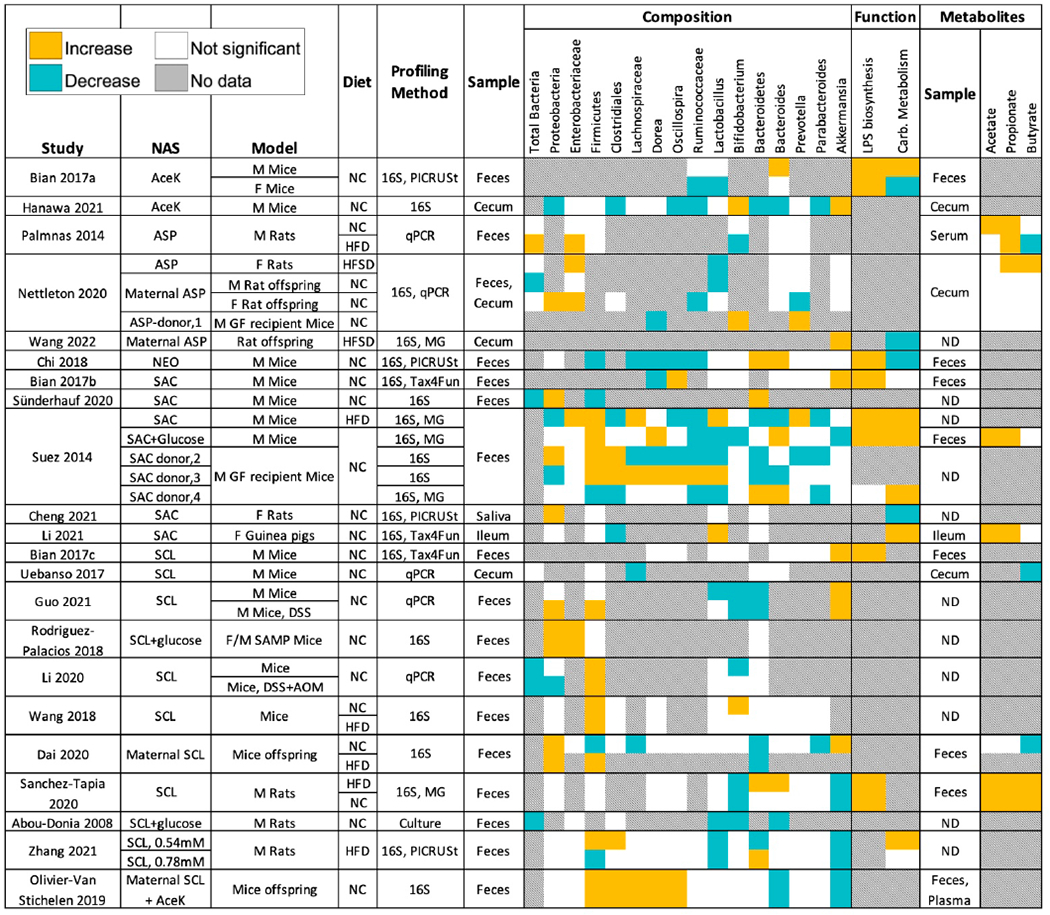
Effects of NAS on the microbiome composition and function. Studies investigating the association of NAS and mammalian microbiomes were retrieved using the search terms (Microbiome OR Microbiota) AND (Saccharin OR Sucralose OR Aspartame OR Acesulfame Potassium OR Neotame) on https://pubmed.ncbi.nlm.nih.gov/. Only research articles were selected. Studies in which the effect of NAS could not be isolated from that of an unrelated additive were excluded from analysis. Microbial features (taxa, functions, metabolites) included in this figure were significantly altered in at least three independent works, regardless of direction of the effect. An indicated feature was labeled as not significantly changed if it was clearly labeled as such in a study, or it was not included in a list reported by the authors as encompassing all significantly altered features. In experiments with dams consuming NAS, pups were exposed prenatally and through lactation, but were not directly supplemented with NAS. AceK, Acesulfame Potassium; ASP, Aspartame; NEO, Neotame; SAC, Saccharin; SCL, Sucralose; NC, Normal Chow; HFD, High Fat Diet; HFSD, High Fat/Sucrose Diet; DSS, Dextran Sulfate Sodium; AOM, Azoxymethane; FMT, Fecal Microbiota Transplant; MG, Metagenomics; GF, Germ-Free; F/M, Female/Male; ND, No Data/Not Determined. 1, FMT from offspring of dams consuming ASP; 2, FMT from mice consuming SAC + glucose; 3, FMT from mice consuming pure SAC and HFD; 4, FMT with fecal microbiome cultured with SAC.

One approach for addressing these mechanistic questions is to identify reproducible NAS-associated microbial signatures. To that aim, we analyzed compositional and functional changes in studies that reported an effect of NAS on the microbiome (Table 1) and plotted the direction of the effect for features (taxa, functional pathways, metabolites) that were significantly altered in at least three independent studies (Figure 1). Despite considerable methodological heterogeneity, several patterns emerge. The abundance of the *Enterobacteriaceae* family (or specifically, *E. coli*) was increased in all studies that reported a significant effect (four studies, six experimental arms). However, while no study reported a significant decrease in *Enterobacteriaceae*, seven studies found no significant change in the abundance of this family. Potentially related to an increase in *Enterobacteriaceae*, the abundance of genes involved in lipopolysaccharide (LPS) biosynthesis was increased in all studies that reported an effect (six studies, nine experimental arms), with only one experimental arm showing no significant effect and no studies reporting a decrease. Despite some variation, a general trend for underrepresentation of Clostridiales associated with butyrate production (*Lachnospiraceae*, *Ruminococcaceae*, *Clostridium* cluster XIVa, *Dorea*, *Oscillospira*) was observed across studies with different NAS, possibly related to the reduction in butyrate observed in three studies. In contrast, abundance of the short-chain fatty acids (SCFA) acetate and propionate was significantly increased in four and five studies, respectively, and no study reported a significant decrease of these two SCFA. Finally, the total number of bacteria (quantified by culture or qPCR) was significantly reduced by diverse NAS in five studies, significantly increased in one, and three studies reported no significant change. These interesting patterns notwithstanding, the key finding of this analysis is the high level of heterogeneity among NAS effects on the microbiome, as even per a given NAS, it was not possible to identify a microbiome feature that was significantly altered in the same direction in all trials. While much of this variation can be attributed to methodological differences, it is reasonable to assume that the interactions of NAS with the microbiome are complex, and an effect on the host may be exerted through more than one mechanism, especially when considering chemically-distinct NAS. Thus, at this stage, an unbiased approach for profiling the microbiome and metabolome of NAS-treated animals would likely be more insightful than focusing on a limited list of microbial features of interest.

Compared to the magnitude of evidence in mammalian models (Table 1, Figure 1), there is a paucity of studies examining interactions between NAS and the microbiome in humans. Two cross-sectional studies found an association between NAS intake and a microbiome signature in individuals consuming aspartame and/or AceK (*N* = 31) [97] or high-consumers of any NAS (*N* = 381) [20], the latter also associated with impaired metabolic health. Maternal consumption of NAS was associated with changes in the infant’s microbiome and a higher BMI (*N* = 100 infants) [98]. In contrast, three interventional studies (with sucralose, *N* = 34; sucralose and aspartame, *N* = 17; or saccharin, *N* = 24) did not find an effect of the above NAS on the microbiome [24,25,35]. Interestingly, results from a small-scale (*N* = 7) saccharin supplementation trial suggest that the effects of this NAS on the microbiome are personalized, as microbiome alterations were more pronounced in a subset of individuals who developed glucose intolerance following saccharin supplementation [20]. As the other intervention trials did not address personalized effects on the microbiome post-NAS supplementation, it is currently unknown whether this preliminary observation is generalizable to other cohorts and other NAS. Clearly, more large-scale RCTs assessing the effect of NAS on both metabolic health and the microbiome are needed. As all of the aforementioned studies profiled the microbiome using 16S rRNA sequencing, the effect of NAS on the human microbiome beyond the genus level, as well as on the microbiome function, remain currently completely unknown.

## PUTATIVE MECHANISMS FOR MICROBIOME MODULATION BY NAS

**Figure 2.**
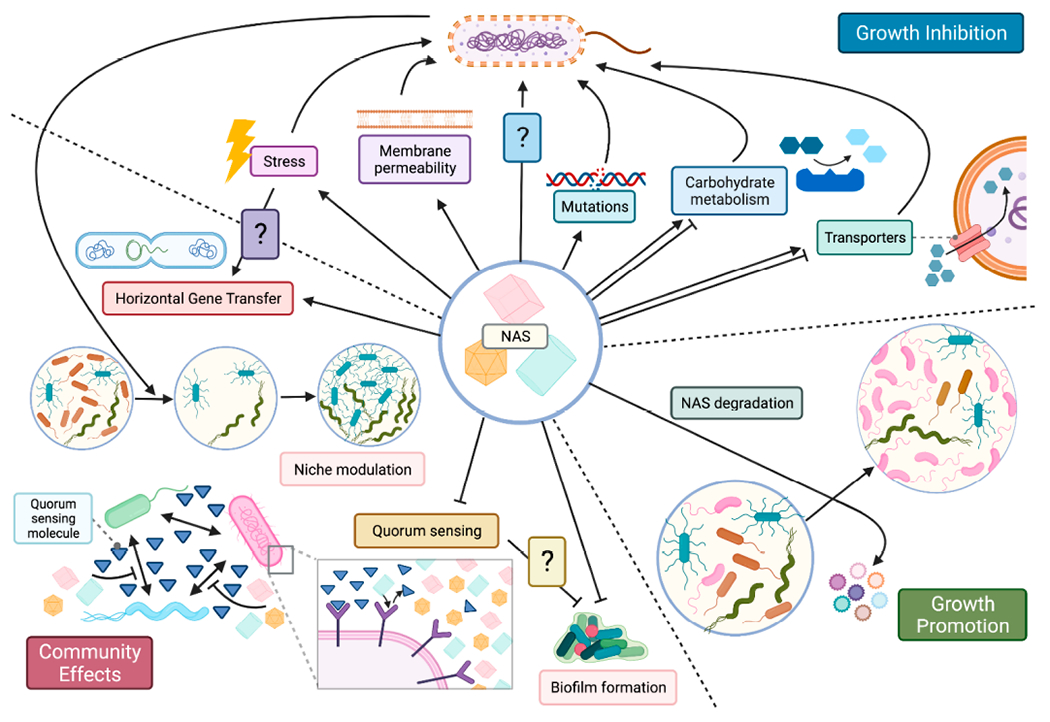
Putative mechanisms for microbiome modulation by NAS. Gut bacteria can directly interact with NAS through several mechanisms, which may lead to growth promotion, inhibition, or community-wide effects.

Alterations in microbiome configuration following exposure to NAS can result from either direct interactions or indirect downstream effects of NAS interactions with the host, such as immune system activation [40,85,86,91]. However, in vitro studies of microbial monocultures or complex microbial communities demonstrate profound effects of NAS on bacterial (and fungal [99]) growth, physiology, metabolism, gene expression, and communication, as well as community-wide effects (Figure 2). Data pertaining to the metabolic fate (pharmacokinetics) of NAS can help shed light on potential direct/indirect mechanisms through which each compound potentially affects the microbiome and the host’s health. Oral administration studies in humans [74], dogs [100], mice [101], and rats [102] indicate that the majority of ingested **sucralose** reaches the large intestine, and a minority is absorbed. Subsequently, the majority (but not all) of sucralose is excreted in feces unchanged, although inter-subject variability was reported [74,100,102,103]. The metabolic fate of the remaining fraction is currently unknown, although sucralose-associated metabolites of unknown function have been identified in urine [74,101,102,104], feces [104], and adipose tissue [104]. These observations suggest that the intestinal microbiome can directly interact with sucralose, and potential metabolism of sucralose by the host and/or microbiome. Unlike sucralose, the majority of orally-administered **saccharin** is slowly absorbed from the gut lumen to the plasma (and consequently excreted in urine) [75,105–108]. Saccharin’s slow absorption, combined with the 5–15% percent that is not absorbed and excreted in feces (and up to 40% in a report in rats [109]), indicate that a non-negligible amount of saccharin may interact with intestinal bacteria. As approximately 99% of ingested saccharin is excreted unchanged, its effects on the microbiome may be mediated through changes in environmental pH or perturbations to carbohydrate metabolism, further discussed below. Somewhat similar to saccharin, the majority of **AceK** administered to rats, dogs, swine, and humans (70–99%) is absorbed and excreted in urine, although inter-subject variability was reported [74,99–102]. Notably, in all dissected animals, the large intestine was a major source of **AceK** (compared to extra-intestinal sites) post-supplementation [110], rendering interactions with the microbiome plausible. Orally administered **cyclamate** is excreted in both urine and feces [111], and metabolism of cyclamate to cyclohexylamine and downstream metabolites occurs in a subject-dependent manner and has been associated with the activity of gut bacteria [112,113]. In contrast to the aforementioned NAS, **aspartame** is broken down to its constituents (methanol, phenylalanine, and aspartate) in the stomach and small intestine. The levels of these metabolites are comparable to those derived from natural ingredients in the human diet [114]. Thus, it is less likely that the effects on the microbiome observed in aspartame-supplemented animals are due to direct interactions of colonic bacteria with aspartame or its derivatives, and the underlying mechanism for microbiome modulation requires further study. One currently untested possibility is that pre-degraded aspartame interacts with bacteria in the most proximal regions of the gastrointestinal tract, namely, the oral cavity, stomach and duodenum, resulting in downstream effects on the colonic microbiome. Alternatively, the effects of aspartame on the microbiome may be host-mediated, for example through interactions with sweet-taste receptors in the gut [115,116]. Oral supplementation with analogs of aspartame, **neotame** and **advantame** [117], results in the majority of the ingested dose excreted in feces as metabolites. Whether these metabolites directly interact with the microbiome is currently unknown.

Several members of the microbiome bloom in the presence of NAS (Figure 1), suggesting a capacity for utilization of NAS as growth substrates, which would confer a competitive advantage in the dense ecological niche of the gut (Figure 2). Enrichment consortia of aerobic environmental bacteria have shown a potential for bacterial saccharin and cyclamate degradation [118]. Aerobic utilization of saccharin as a sole carbon source was observed in a sewage isolate of *Sphingomonas xenophaga* [119]. *Lactobacillus delbrueckii* isolated from commercial yogurt could utilize aspartame as a carbon source [120], and several oral auxotrophic bacteria demonstrated an ability to utilize aspartame as a source for phenylalanine in vitro, suggesting catabolic capacity [121]. Uptake of NAS may depend upon external factors; for example, *Streptococcus mutans* saccharin uptake in culture is contingent upon the co-occurrence of glycolysis and an acidic extracellular pH [122]. These studies indicate that some bacteria possess the enzymatic machinery required to degrade man-made NAS. However, the abundance of these metabolic pathways in the gut and whether such uptake and utilization occur in vivo remain to be determined.

In contrast to a growth-promoting effect, and in line with the observation that NAS are associated with a reduction in bacterial load in vivo (Figure 1) [38,78,79,93,94], multiple in vitro studies demonstrate that NAS can directly inhibit bacterial growth (Figure 2) [79,88,123–129]. Some of these effects may stem from NAS impacts on bacterial carbohydrate metabolism (Figure 2). Saccharin may interfere with microbial glucose transport, metabolism, and fermentation [126,129–131] and was shown to modify expression of glucose transport and metabolism genes in *Lactobacillus* [84]. A bacteriostatic effect of sucralose was associated with its ability to decrease sucrose uptake and competitively inhibit enzymatic sucrose degradation [125]. Notably, multiple studies have demonstrated an impact of NAS on abundance of genes related to carbohydrate metabolism (Figure 1) [20,39,77,80,92] and transport [20,84,125,131] in vivo. The degree of growth inhibition may vary between bacterial species [79,128,130], and species-specific impacts of inhibition could therefore alter microbiome composition and contribute to dysbiosis in vivo [79]. Thus, the bloom of gut bacteria may be a result of expansion into a niche previously occupied by NAS-inhibited bacteria (Figure 2) rather than a direct growth-promoting effect.

NAS may impact other microbial functions beyond metabolism. Aspartame, sucralose, and saccharin can inhibit gut bacterial quorum sensing, possibly through interfering with ligand binding of the LasR receptor [132]. Reduced levels of quorum sensing autoinducers have also been observed in fecal metabolomes of mice consuming sucralose [40]. NAS have also been found to increase cell envelope permeability and stimulate expression of DNA translocation machinery, with the potential to promote increased rates of horizontal gene transfer [133,134]. In *Enterococcus faecalis* and *E. coli*, the aforementioned three NAS could increase bacterial biofilm formation and capacity to adhere to and kill human gut epithelium in vitro [135]. Intriguingly, this effect was blocked in vitro by the pan-sweet taste inhibitor zinc sulphate. Aspartame has also been shown to increase prophage induction in *E. faecalis* [136], while sucralose increases the mutation rate of *E. coli* in vitro [137]: both likely indicate activation of microbial stress response to NAS. Consistent with evidence for bacterial stress response, reactive oxygen species (ROS) and SOS-related stress genes are upregulated in response to AceK [134], and NAS-mediated induction of cellular stress was demonstrated in *E. coli* [129]. Thus, NAS appear to have several indirect effects on bacterial social behavior that may further alter gut microbiome dynamics through impacts on microbe-microbe interactions (Figure 2). Collectively, NAS may affect multiple bacterial targets, resulting in direct community modulation (Figure 2). While host-mediated indirect effects of NAS on the microbiome cannot be ruled out, direct alteration of a complex fecal microbiome community by saccharin in vitro was demonstrated to be sufficient for promoting glucose intolerance in recipient GF mice [20]. Identifying bacteria directly impacted by NAS could potentially serve as a useful marker for predicting metabolic responsiveness of humans to NAS and can facilitate understanding of microbial functions that negatively impact metabolic health of the host.

### Putative Mechanisms for Modulation of Host Metabolic Health by NAS-Associated Microbiome

To date, a causal link between NAS-associated microbiomes and a negative impact on metabolic health has been established in GF mouse recipients of the following fecal microbiomes: saccharin-treated mice (pure or in combination with glucose) [20]; human responders to saccharin [20]; treated in vitro with saccharin [20]; and pups of aspartame-drinking dams [78]. The underlying mechanisms, however, are currently poorly understood. Of the various effects of NAS on the microbiome function or its associated metabolome, two patterns appear more consistent than others (Figure 1) and are worth discussing: an increase in the abundance of LPS biosynthesis genes [20,40,77,91] and an increase in the abundance of the SCFA acetate and propionate [20,48,91,94]. Notably, while no study has reported a significant opposite trend, some studies found no significant effect of NAS on LPS or SCFA. Thus, even if they do mediate an effect of the NAS-associated microbiome on metabolic health, they are likely only part of a more complex interaction.

Overrepresentation of LPS biosynthesis genes could potentially contribute to impaired metabolic health through a process termed “metabolic endotoxemia”, which has been linked with obesity and insulin resistance in mice and humans [138–140]. In this process, disrupted intestinal barrier function (resulting from modulation of the microbiome by diet, or LPS itself) leads to chronically elevated plasma levels of microbial-associated molecular patterns, predominantly LPS. The result is a TLR4- and CD14-dependent systemic and tissue-specific low-grade inflammation, including the adipose, liver, and skeletal muscle tissues. In addition to increased abundance of LPS biosynthetic genes, several studies in animal models have associated saccharin or sucralose supplementation with an increase in inflammatory markers [40,76,85–87,90,91,94] and modulation of intestinal barrier permeability [85,90,91,94], and one study specifically assessed the effect of sucralose on components of metabolic endotoxemia [91]. More conclusive evidence is needed to decipher the contribution of this mechanism to NAS-associated metabolic derangements, for example in TLR4-deficient animals.

SCFA are key modulators of host-microbiome interactions and serve as signaling molecules, either by inhibiting histone deacetylases (HDACs) or by acting as ligands for several G protein-coupled receptors (GPR41, GPR43, GPR109A) and peroxisome proliferator-activated receptor-γ (PPARγ) [141,142]. In the context of metabolic health, higher cecal levels of acetate and butyrate were reported in obese mice [143], and fecal propionate was elevated in humans [144] with obesity. Consumption of a diet rich in saturated fat was associated with increased fecal levels of acetate, propionate, and butyrate in individuals with metabolic syndrome [145], and weight loss was associated with reduced plasma propionate in humans [146]. Dietary supplementation of propionate to mice and humans was shown to disrupt glucose tolerance [146], and high-fat diet feeding to rats resulted in increased microbial production of acetate, which led to overproduction of insulin and the hunger-associated hormone ghrelin, resulting in hyperphagia and obesity [147]. Conflictingly, multiple other studies [142] have reported improvement of metabolic health associated with supplementation of acetate [148,149], propionate [53,150–152], or butyrate [53,152,153], suggesting the need for a more refined understanding of tissue specificity and the distinction between endogenous production and supplementation. Whether acetate, propionate, and butyrate mediate a negative impact of NAS on metabolic health, or rather are an unrelated outcome of microbiome modulation, remains to be determined.

## PERSPECTIVE: THE IMPORTANCE OF PRECISION

The major challenge pertaining to the study of NAS remains a public health one, i.e., determining the extent to which NAS might negatively affect metabolic health. While the literature remains inconclusive, several health authorities and organizations recommend a cautious approach; Canada [154] and Israel [155] recommend overall reduction of sweeteners, caloric or not; the European Union restricts NAS in infant food [156] and in baked goods marketed towards individuals with diabetes [157], and the American Heart Association has advised against NAS consumption amongst children due to the unknown long-term effects [4]. Identifying correlates of responsiveness to NAS could help distinguish between scenarios in which NAS pose a health risk versus those in which they may be consumed safely, an important consideration, particularly for individuals with diabetes.

The major extrinsic factor to consider is the type of NAS and whether some pose a greater risk than others. This level of precision is not feasible in observational studies, in which participants who consume NAS-containing products are exposed to multiple different types of NAS in their habitual diet, sometimes unknowingly. Distinguishing between effects of specific NAS could be achieved with NAS supplementation to NAS-abstainers. Currently, there is insufficient evidence in humans that would allow meta-analyses of RCTs to stratify metabolic outcomes per a given NAS. Additional RCTs, especially those that perform head-to-head comparisons of different NAS, are critically required.

In addition to the intervention itself, a precision approach to health and nutrition necessitates consideration of individual-specific factors, including age, sex, medical history, habitual diet, and the intestinal microbiome [70]. An important factor in precision nutrition [61,65,70], preliminary evidence indicates that the microbiome may predict and modulate human metabolic responses to NAS [20]. Three other interventional studies that examined the effects of NAS on the microbiome did not address personalized post-exposure differences, and therefore it is currently unknown whether this finding is generalizable and applies to all NAS. Nonetheless, the magnitude of evidence for an effect of NAS on the microbiome in animal models should encourage further pursuit of similar impacts in humans.

Exploration of the mechanisms through which NAS interact with the microbiome in modulation of the host’s metabolic health is still in its early stages. Multiple potential mechanisms through which each NAS can affect microbial physiology have been described predominantly in vitro (Figure 2) and remain to be validated in vivo and in humans. Moving beyond associations, studies that causally link microbiome modulation by NAS with an effect on host metabolism [20,78] can serve as a powerful tool for deciphering the underlying mechanisms. While some mechanistic links are more common than others (namely, SCFA production and metabolic endotoxemia, Figure 1), the lack of a consistent signature even within a single NAS suggest that these may be an outcome, rather than the driver, of the metabolic phenotype, which could also explain how the same pathways manifest after supplementation with chemically-distinct NAS. Indeed, hyperglycemia may promote impaired barrier function [158], and elevated SCFA levels have been suggested to be a marker, rather than a driver, of metabolic syndrome [142]. Thus, in addition to establishing causality (e.g., using transplantation to GF animals), future research would benefit from a longitudinal approach that would enable investigators to decipher the sequence of events.

Heterogeneity in the microbiome of NAS-exposed individuals could conduce to differential metabolic outcomes if the abundances of microbes amenable to modulation by NAS displays person-to-person variation. Such heterogeneity may also underlie inter-subject differences in excretion and absorption reported for some NAS [74,75,100–117,159] and thus conduce differential effects on the host by controlling the levels of NAS and, for some, their potential metabolites. Beyond providing critical evidence of causality, understanding the mechanisms involved could provide quantifiable bio-markers of responsiveness to NAS, which could be used in precision nutrition and clinical decision-making.

## Figures and Tables

**Table 1.** Studies examining NAS-microbiome interactions in mammalian model systems. Studies investigating the association of NAS and mammalian microbiomes were retrieved using the search terms (Microbiome OR Microbiota) AND (Saccharin OR Sucralose OR Aspartame OR Acesulfame Potassium OR Neotame) on https://pubmed.ncbi.nlm.nih.gov/. Only research articles were selected. Studies in which the effect of NAS could not be isolated from that of an unrelated additive were excluded from analysis. Studies were analyzed for sweetener used, model system, diet, length of diet and NAS administration, NAS dose, experimental controls, profiling method, effects on the host microbiome, and effects on the host metabolic phenotype.

Study	NAS	Model	Diet	NAS Dose/Concentration	Control	Profiling Method	Microbiome	Metabolic Phenotype
Bian 2017 [39]	AceK	M Mice	4 weeks NC, NAS by gavage	37.5 mg/kg/day	Water	16S, PICRUSt	Yes	Yes
Bian 2017 [39]	AceK	F Mice	4 weeks NC, NAS by gavage	37.5 mg/kg/day	Water	16S, PICRUSt	Yes	Yes
Hanawa 2021 [93]	AceK	M Mice	8 weeks NC, NAS in water	150 mg/kg/day	Water	16S (cecum)	Yes	ND
Uebanso 2017 [52]	AceK	M Mice	8 weeks NC, NAS in water	15 mg/kg/day	Water	16S (cecum)	No	No
Wang 2021 [96]	Aspartame	F Rats	10 weeks HFSD, 6 weeks NAS in water	40 mg/kg/day	Water	16S (cecum)	No	No
Wang 2021 [96]	Aspartame	M/F Rat offspring to ASP-consuming obese dams	3 weeks MM, 15 weeks NC	40 mg/kg/day (dams only)	Offspring to water dams	MG, 16S (cecum)	Yes	Yes
Palmnas 2014 [48]	Aspartame	M Rats	8 weeks NC, NAS in water	5–7 mg/kg/day	Water	16S	Yes	Yes
Palmnas 2014 [48]	Aspartame	M Rats	8 weeks HFD, NAS in water	5–7 mg/kg/day	Water	16S	Yes	Yes
Nettleton 2020 [78]	Aspartame	F Rats	16 weeks HFSD, 6 weeks NAS in water	7 mg/kg	Water	qPCR (cecum)	Yes	Yes
Nettleton 2020 [78]	Aspartame	M Rat offspring to ASP-consuming dams	3 weeks MM, 15 weeks NC	7 mg/kg (dams only)	Offspring to water dams	qPCR (cecum)	Yes	Yes
Nettleton 2020 [78]	Aspartame	F Rat offspring to ASP-consuming dams	3 weeks MM, 15 weeks NC	7 mg/kg (dams only)	Offspring to water dams	qPCR (cecum)	Yes	Yes
Nettleton 2020 [78]	Aspartame	M GF recipient Mice (FMT from M Rat offspring to ASP-consuming dams)	NC 15 days post-FMT	7 mg/kg (donors’ dams)	FMT from offspring to water dams	qPCR (cecum)	Yes	Yes
Chi 2018 [77]	Neotame	Mice	4 weeks NC, NAS by gavage	0.75 mg/kg/day	Water	16S	Yes	ND
Bian 2017 [76]	Saccharin	M Mice	6 months NC, NAS in water	0.3 mg/mL	Water	16S	Yes	ND
Suez 2014 [20]	Saccharin + glucose	M Mice	11 weeks NC, NAS in water	5 mg/mL with 95 mg/mL glucose	Glucose/Sucrose/Water	16S, MG	Yes	Yes
Suez 2014 [20]	Saccharin	M Mice	5 weeks HFD, NAS in water	0.1 mg/mL	Water	16S, MG	Yes	Yes
Suez 2014 [20]	Saccharin + glucose	M GF recipient Mice (FMT from saccharin-consuming mice)	NC 6 days post-FMT	5 mg/mL with 95 mg/mL glucose (donor)	FMT from glucose-drinking mice	16S	Yes	Yes
Suez 2014 [20]	Saccharin	M GF recipient Mice (FMT from HFD saccharin-consuming mice)	NC 6 days post-FMT	0.1 mg/mL (donor)	FMT from HFD water-drinking mice	16S	Yes	Yes
Suez 2014 [20]	Saccharin	M GF recipient Mice (FMT from microbiome cultured w/ SAC)	NC 6 days post-FMT	5 mg/mL (donor culture SAC concentration)	FMT from microbiome cultured with PBS	16S, MG	Yes	Yes
Sünderhauf 2020 [79]	Saccharin	Mice	5 weeks NC, NAS in water	0.1 mg/mL	Water	qPCR, 16S	Yes	ND
Serrano 2021 [35]	Saccharin	Mice	10 weeks NC, NAS in water	250 mg/kg/day	Water	16S	No	No
Serrano 2021 [35]	Saccharin	T1R2-KO Mice	10 weeks NC, NAS in water	250 mg/kg/day	Water	16S	No	No
Cheng 2021 [80]	Saccharin	F Rats	8 weeks NC, NAS in water	0.83 mg/mL	Water/0.83 mg/mL sucrose	16S (oral), PICRUSt	Yes	ND
Anderson 1980 [81]	Saccharin	M Rats	10 days NC, NAS in diet	7.5% w/w	7.5% w/w cellulose	Culture	Yes	ND
Falcon 2020 [82]	Saccharin	Rats	17 weeks, NAS in yogurt	0.3% w/w	20% sucrose yogurt	16S	No	ND
Lyte 2016 [83]	Saccharin	Selectively bred high saccharin intake Rats	3 days NC, 1 day NAS in water	0.1% w/v	Selectively bred low saccharin intake Rats	16S	Yes	Yes
Li 2021 [89]	Saccharin	F Guinea Pigs	4 weeks NC, NAS in water	1.5 mM	Water	16S, Tax4Fun	Yes	Yes
Daly 2016 [84]	Saccharin + NHDC	Piglets	2 weeks, NAS in diet	0.015% w/w SUCRAM	NC	16S	Yes	ND
Bian 2017 [40]	Sucralose	M Mice	6 months NC, NAS in water	0.1 mg/ml	Water	16S, Tax4Fun	Yes	Yes
Guo 2021 [85]	Sucralose	M Mice	6 weeks NC, NAS in water	1.5 mg/mL	Water	qPCR	Yes	ND
Guo 2021 [85]	Sucralose	M Mice w/DSS-induced colitis	6 weeks NC, NAS in water	1.5 mg/mL	Water w/DSS-induced colitis	qPCR	Yes	ND
Uebanso 2017 [52]	Sucralose	M Mice	8 weeks NC, NAS in water	1.5 mg/kg/day	Water	qPCR (cecum & feces)	No	No
Uebanso 2017 [52]	Sucralose	M Mice	8 weeks NC, NAS in water	15 mg/kg/day	Water	qPCR (cecum & feces)	Yes	Yes
Rodriguez-Palacios 2018 [86]	Sucralose	F/M SAMP Mice	6 weeks NC, NAS in water	3.5 mg/mL	Water	16S	Yes	Yes
Rodriguez-Palacios 2018 [86]	Sucralose	F/M AKR Mice	6 weeks NC, NAS in water	3.5 mg/mL	Water	16S	Yes	No
Li 2020 [94]	Sucralose	Mice	11 weeks NC, NAS in water	1.5 mg/mL	Water	16S	Yes	ND
Li 2020 [94]	Sucralose	Mice w/AOM/DSS-	11 weeks NC, NAS in water	1.5 mg/mL	Water w/AOM/DSS	16S	Yes	ND
Martinez-Carillo 2019 [87]	Sucralose + glucose	Mice	6 weeks NC, NAS in water	4.1 mg/mL Splenda® for 5 h/day	41.66 mg/mL sucrose, water	16S (small intestine)	Yes	Yes
Martinez-Carillo 2019 [87]	Sucralose + glucose	Mice	12 weeks NC, NAS in water	4.1 mg/mL Splenda® for 5 h/day	41.66 mg/mL sucrose, water	16S (small intestine)	Yes	Yes
Wang 2018 [88]	Sucralose	Mice	8 weeks HFD, NAS in water	2.5% w/v	Water	16S	Yes	No
Wang 2018 [88]	Sucralose	Mice	8 weeks NC, NAS in water	2.5% w/v	Water	16S	Yes	No
Dai 2020 [90]	Sucralose	Mice offspring to SCR-consuming dams	3 weeks MM	0.1 mg/mL (dams only)	Offspring to water dams	16S	Yes	Yes
Dai 2020 [90]	Sucralose	Mice offspring to SCR-consuming dams	3 weeks MM, 5 weeks NC, 4 weeks HFD	0.1 mg/mL (dams only)	Offspring to water dams	16S	Yes	Yes
Dai 2021 [95]	Sucralose	Mice offspring to SCR-consuming dams	3 weeks MM	0.1 mg/mL (dams only)	Offspring to water dams	16S	Yes	Yes
Dai 2021 [95]	Sucralose	Mice offspring to SCR-consuming dams	3 weeks MM, 5 weeks NC	0.1 mg/mL (dams only)	Offspring to water dams	16S	Yes	ND
Sanchez-Tapia 2020 [91]	Sucralose	M Rats	4 months HFD, NAS in water	1.5% in water	Water	16S, MG	Yes	Yes
Sanchez-Tapia 2020 [91]	Sucralose	M Rats	4 months NC, NAS in water	1.5% in water	Water	16S, MG	Yes	Yes
Abou-Donia 2008 [38]	Sucralose + glucose	M Rats	12 weeks NC, NAS by gavage	100 mg/kg/day Splenda®	Water	Culture + plate counts	Yes	Yes
Abou-Donia 2008 [38]	Sucralose + glucose	M Rats	12 weeks NC, NAS by gavage	300 mg/kg/day Splenda®	Water	Culture + plate counts	Yes	No
Abou-Donia 2008 [38]	Sucralose + glucose	M Rats	12 weeks NC, NAS by gavage	500 mg/kg/day Splenda®	Water	Culture + plate counts	Yes	Yes
Abou-Donia 2008 [38]	Sucralose + glucose	M Rats	12 weeks NC, NAS by gavage	1000 mg/kg/day Splenda®	Water	Culture + plate counts	Yes	No
Zhang 2021 [92]	Sucralose	M Rats	12 weeks HFD, 4 weeks NAS by gavage	0.54 mM in water	Water, 324 mM sucrose	16S, PICRUSt	Yes	ND
Zhang 2021 [92]	Sucralose	M Rats	12 weeks HFD, 4 weeks NAS by gavage	0.78 mM in water	Water, 324 mM sucrose	16S, PICRUSt	Yes	ND
Olivier-Van Stichelen 2019 [46]	Sucralose & AceK	Mice offspring to SCR + AceK-consuming dams	19 days MM	0.1 mg SCR + 0.25 mg AceK (dams only)	Offspring to water dams	16S	Yes	Yes
Olivier-Van Stichelen 2019 [46]	Sucralose & AceK	Mice offspring to SCR + AceK-consuming dams	19 days MM	0.2 mg SCR + 0.5 mg AceK (dams only)	Offspring to water dams	16S	Yes	Yes

AceK, Acesulfame Potassium; NC, Normal Chow; HFD, High Fat Diet; MM, Maternal Milk; HFSD, High Fat/Sucrose Diet; DSS, Dextran Sulfate Sodium; AOM, Azoxymethane; NHDC, neohesperidin dihydrochalcone; FMT, Fecal Microbiota Transplant; MG, Metagenomics; GF, Germ-Free; F/M, Female/Male; ND, No Data/Not Determined.
